# S-Box Design Based on 2D Multiple Collapse Chaotic Map and Their Application in Image Encryption

**DOI:** 10.3390/e23101312

**Published:** 2021-10-06

**Authors:** Chao Yang, Xia Wei, Cong Wang

**Affiliations:** School of Electrical Engineering, Xinjiang University, Urumqi 830046, China; yycc367@stu.xju.edu.cn (C.Y.); cw1989@st.btbu.edu.cn (C.W.)

**Keywords:** 2D chaotic map, chaotic S-box, diffusion, image encryption

## Abstract

As an essential part of an encryption system, the performance of a chaotic map is critical for system security. However, there are many defects for the existing chaotic maps. The low-dimension (LD) ones are easily predicted and are vulnerable to be attacked, while high-dimension (HD) ones have a low iteration speed. In this paper, a 2D multiple collapse chaotic map (2D-MCCM) was designed, which had a wide chaos interval, a high complexity, and a high iteration speed. Then, a new chaotic S-box was constructed based on 2D-MCCM, and a diffusion method was designed based on the S-box, which improved security and efficiency. Based on these, a new image encryption algorithm was proposed. Performance analysis showed that the encryption algorithm had high security to resist all kinds of attacks easily.

## 1. Introduction

With the rapid development of the network, image transmission through network has become more and more popular, contributing to higher risk of information leakage [1]. Therefore, the security of image transmission has become a research hotspot. Encryption of an image is the most direct and effective way to ensure image security [2,3]. In addition, as a large amount of information can be carried by images, higher speed of encryption algorithm is required. Because chaotic maps can quickly generate highly complex pseudo-random sequences, the combination of chaotic map and image encryption has become a focus of attention [4,5,6].

The research of image encryption algorithm based on chaotic maps is mainly focused on the optimization algorithm structure and optimization of chaotic map. For the former one, common encryption algorithms consist of two steps: scrambling and diffusion. Scrambling can not only change the pixel position in the image but also destroy the image structure. For image encryption algorithms based on chaotic maps, it is most common to directly scramble the image by using the index of chaotic sequence [7]. With further research, some other scrambling methods have been designed such as wavelet transform [8,9], cellular automata [10,11], and special matrix [12,13,14].

Diffusion is particularly important for encryption algorithms, which can extend the local changes in the image to the whole and, finally, change the pixels in the image and hide the image information. In addition to using chaotic sequences for diffusion [15], some methods in other fields, such as DNA computing [16,17,18] and Brownian motion [19,20], also help in diffusion. However, the implementation of the methods mentioned above is relatively complex. For example, encryption using DNA computing requires definitions of DNA addition and subtraction, which make the implementation far more difficult and, thus, make the speed of encryption slower. However, the substitution box (S-box), as a square matrix, becomes a key part of the block cipher, achieving a non-linear transformation of the input, and it is widely used because of high speed and security [21,22,23,24,25,26].

The optimization of chaotic maps is another research hotspot. The performance of chaotic maps will directly affect the security of encryption algorithms, and some encryption algorithms based on simple chaotic maps have been proven to be insecure. There are two main methods to search for high performance chaotic maps: to explore the combination or variation of existing chaotic maps [15,27,28] and to construct new chaotic maps [7,29,30,31]. Essentially, both approaches in many cases are the explorations of higher-dimensional chaotic maps. HD chaotic map has many advantages, such as more parameters and variables and high complexity, but there are some disadvantages, such as low iteration speed and unstable chaotic state. Therefore, it is vital to design a chaotic map that can provide a high complexity chaotic sequence without much computation.

Therefore, we proposed a new two-dimensional (2D) chaotic map, called 2D multiple collapse chaotic map (2D-MCCM). Based on the analysis of its phase trajectory, bifurcation diagram, Lyapunov exponent, and entropy spectrum, it is demonstrated that, compared with the existing 2D chaotic maps, 2D-MCCM has stronger randomness, larger chaotic range, and higher complexity. Then, a high-performance S-box was designed based on 2D-MCCM and the self-scrambling method. The S-box can be applied in diffusion and can obtain efficient and remarkable results. Therefore, we proposed an image encryption algorithm based on 2D-MCCM and S-box. With S-box, the security and efficiency of the algorithm can be dramatically improved.

The rest of this paper is organized as follows. Section 2 presents a 2D-MCCM chaotic map and its performance. Section 3 introduces the generation method of S-boxes and analyzes the security of the generated S-boxes. An image encryption algorithm based 2D-MCCM and S-boxes is presented in Section 4. Section 5 evaluates the performance of the encryption algorithm. Finally, Section 6 concludes the paper.

## 2. Design of 2D Chaotic Map

Stretching and collapse are the conditions of chaos behavior in the map. Stretching will lead to the separation of adjacent phase points, which reflects the sensitivity of the initial value of the map. Collapse is to constrain the map to a fixed region, so every *x* corresponds to multiple *y*, and, thus, chaos is generated accordingly.

For map *f* (*x*) and *g* (*x*), and assuming that *f*′(*x*) ≥ *g*′(*x*), *x* ∈ [*a*, *b*], it can be obtained as

$$\begin{array}{l} \;f^{\prime }(x)-g^{\prime }(x)\geq 0\\ \Rightarrow f(b)-g(b)\geq f(a)-g(a)\\ \Rightarrow f(b)-f(a)\geq g(b)-g(a) \end{array}$$

If *f* (*x*) and *g* (*x*) are collapsed into [*c*, *d*], where *c* ≥ *a*, *d* ≤ *b*, it can be obtained as

$$\frac{f(b)-f(a)}{d-c}\geq \frac{g(b)-g(a)}{d-c}$$

Based on the Lyapunov exponent [32] definition equation that

(1) $$\lambda =\underset{n\to \infty }{\lim}\frac{1}{n}\ln|\prod_{i=0}^{n-1}F^{\prime }(x_{i})|.$$

Presuming that *f*′(*x*) ≥ *g*′(*x*), so

(2) $$\begin{array}{l} \;\left|\prod_{i=0}^{n-1}f^{\prime }(x)\right|\geq \left|\prod_{i=0}^{n-1}g^{\prime }(x)\right|\\ \Rightarrow \underset{n\to \infty }{\lim}\frac{1}{n}\ln\left|\prod_{i=0}^{n-1}f^{\prime }(x)\right|\geq \underset{n\to \infty }{\lim}\frac{1}{n}\ln\left|\prod_{i=0}^{n-1}g^{\prime }(x)\right|\\ \Rightarrow \lambda_{f}\geq \lambda_{g} \end{array}$$

It indicates that the larger the derivative of the map is, the more times it is collapsed and the greater the Lyapunov exponent is.

Some simple one-dimensional (1D) chaotic maps can be generated by stretching and collapse. Currently, maps are collapsed mainly by adding function segments, trigonometric functions, and modular operations. For example, the Chebyshev map is achieved with the use of the cosine function to collapse the monotonic subtraction function arccos(*x*) to [−1, 1], resulting in chaotic behavior. Similarly, the Iterative chaotic map with infinite collapses (ICMIC) [33] is achieved with the use of sinusoidal functions to collapse the monotone decreasing function (1/*x*) to [−1, 1]. Additionally, Tent Map uses piecewise functions to double the range of functions before they collapse back to the original range. However, these 1D chaotic map structures are too simple, so their chaotic behavior can be easily predicted and the encryption algorithm is not secure when they are used.

### 2.1. Definition of 2D-MCCM

In order to solve the problems above, we designed a new 2D Multiple Collapse Chaotic Map (2D-MCCM). It has much more complex chaotic behavior and higher iteration speed than a 1D chaotic map, which is what HD chaotic maps do not have. The mathematical expression of 2D-MCCM is as follows:

(3) $$\left\{\begin{array}{l} x_{n+1}=\arctan\left[\frac{b}{10ay_{n}}+\tan(a\pi x_{n})\right];\\ y_{n+1}=\arctan\left[\frac{b}{ax_{n}}+\tan(10a\pi y_{n})\right]. \end{array}\right.$$

where, *a* and *b* are parameters, *a*, *b* ∈ (−∞, +∞).

In 2D-MCCM, the arctangent function is used to collapse the map instead of the sine function. Although the sine function can collapse the map to [−1, 1] and collapse the same map several times, the arctangent function as a monotone function can make the distribution of chaotic sequence more uniform. Moreover, the chaotic map has a wider chaotic domain, which makes it more suitable for image encryption.

### 2.2. Performance Evaluation

To evaluate the performance of 2D-MCCM, we use chaotic trajectories, bifurcations, Lyapunov exponents, and permutation entropy to analyze the attractors, chaotic sequence distribution, initial value sensitivity, and system complexity of 2D-MCCM, and compare them with four recently proposed 2D chaotic maps used for image encryption, i.e., 2D Sine Logistic modulation map (2D-SLMM) [34], 2D Logistic ICMIC cascade map (2D-LICM) [35], 2D Logistic-Sine-Coupling Map (2D-LSCM) [15], and 2D infinite collapse map (2D-ICM) [7], which are defined as follows.


(4) $$\begin{array}{l} \text{2D-SLMM: } \end{array}\left\{\begin{array}{c} x_{i+1}=a(\sin(\pi y_{i})+b)x_{i}(1-x_{i});\\ y_{i+1}=a(\sin(\pi x_{i+1})+b)y_{i}(1-y_{i}). \end{array}\right.$$

(5) $$\begin{array}{l} \text{2D-LICM: } \end{array}\left\{\begin{array}{c} x_{i+1}=\sin(21/(b(y_{i}+3)ax_{i}(1-ax_{i})));\\ y_{i+1}=\sin(21/(b(ax_{i+1}+3)y_{i}(1-y_{i}))). \end{array}\right.$$

(6) $$\begin{array}{l} \text{2D-LSCM:} \end{array}\left\{\begin{array}{c} x_{i+1}=\sin(\pi (4ax_{i}(1-x_{i})+(1-a)\sin(\pi y_{i})));\\ y_{i+1}=\sin(\pi (4ay_{i}(1-y_{i})+(1-a)\sin(\pi x_{i+1}))). \end{array}\right.$$

(7) $$\begin{array}{l} \text{2D-ICM: } \end{array}\left\{\begin{array}{c} x_{i+1}=\sin(\frac{a}{y_{i}})\cdot \sin(\frac{b}{x_{i}});\\ y_{i+1}=\sin(\frac{a}{x_{i}})\cdot \sin(\frac{b}{y_{i}}). \end{array}\right.$$

#### 2.2.1. Phase Trajectory

**Figure 1:**
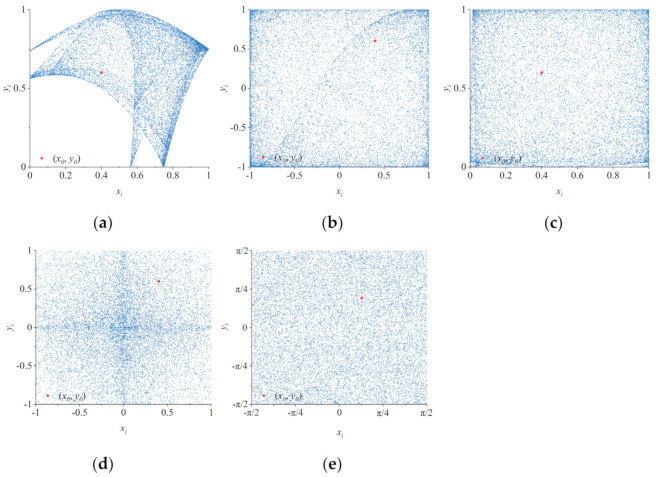
Phase trajectory of five 2D chaotic maps: (**a**) the 2D-SLMM with parameter (*a*, *b*) = (1, 3); (**b**) the 2D-LICM with parameter (*a*, *b*) = (0.8, 0.6); (**c**) the 2D-LSCM with parameter *a* = 0.99; (**d**) the 2D-ICM with parameter (*a*, *b*) = (2, 21); (**e**) the 2D-MCCM with parameter (*a*, *b*) = (0.2, 10).

Chaotic motion is an aperiodic reciprocating motion whose phase trajectory is a curve that never closes and is confined to a bounded region and, thus, leads to the generation of strange attractors. The dynamic characteristics of a chaos map can be preliminarily described based on its phase trajectory diagram. In general, for chaotic maps, the larger the distribution of strange attractors in the phase space is, the more uniform the distribution is, and the better randomness the chaotic sequence will have. In order to analyze the performance of 2D-MCCM, we set the initial values (*x_0_*, *y_0_*) = (0.4, 0.6) of 2D-MCCM, and took four 2D maps as comparison and iterated them for 20,000 times. In order to obtain the chaotic sequence in stable state, only the last 15,000 terms were selected for the phase trajectory diagram, as shown in Figure 1.

As shown in Figure 1, the trajectories of 2D-LICM, 2D-ICM, and 2D-MCCM all fill the whole phase space. However, it is obvious that the phase space of 2D-MCCM is larger and the trajectory distribution is more uniform. This indicates that the chaotic sequence generated by 2D-MCCM iteration has stronger randomness and its results are more difficult to be predicted, so it has higher security when used for image encryption.

#### 2.2.2. Bifurcation Diagram

**Figure 2:**
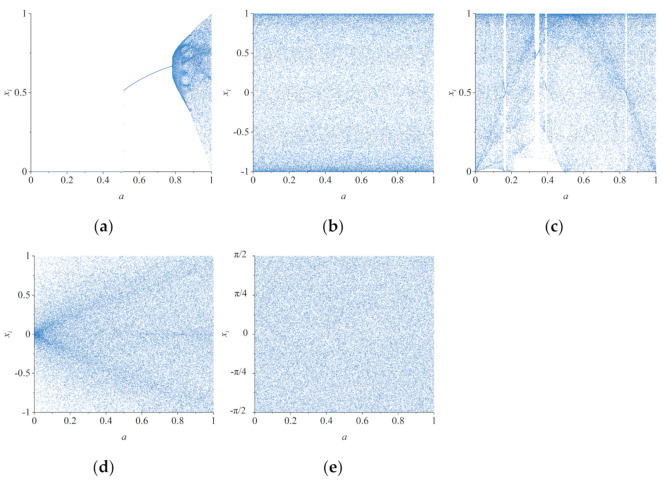
Bifurcation diagram of five 2D chaotic maps: (**a**) 2D-SLMM; (**b**) the 2D-LICM with parameter *b* = 0.5; (**c**) 2D-LSCM; (**d**) 2D-LSCM with parameter *b* = 21; (**e**) the 2D-MCCM with parameter *b* = 21.

**Figure 3:**
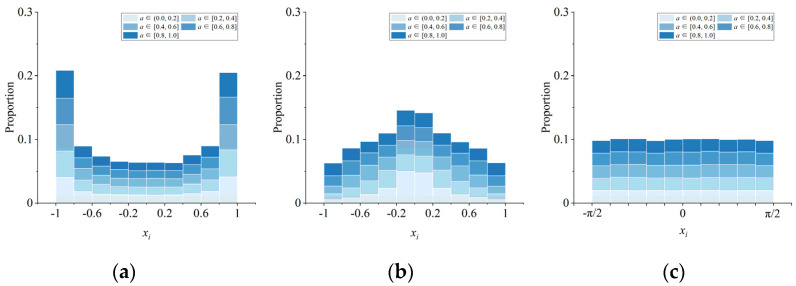
Distribution of points in bifurcation diagrams: (**a**) 2D-LICM; (**b**) 2D-ICM; (**c**) 2D-MCCM.

A bifurcation diagram can show how the variables of a chaotic system vary with bifurcation parameters, which is similar to phase trajectory diagram. The more uniform the distribution of bifurcation diagram is, the stronger the randomness of the chaotic system is. The difference is that the phase trajectory diagram only shows the trajectory of the chaotic map under certain parameter values, while the bifurcation diagram can show how the chaotic system behaves with the change of parameters. Figure 2 shows the bifurcation diagram of *x* component in 2D-MCCM and four 2D maps, respectively. The initial value is set as (*x_0_*, *y_0_*) = (0.4, 0.6). Since parameter *a* in 2D-LICM and 2D-ICM is not equal to 0, the parameter field is set as (0, 1]. It can be seen that only the bifurcation diagrams of 2D-LICM, 2D-ICM, and 2D-MCCM cover the entire chaotic interval. In order to describe the distribution of points in the bifurcation diagram more intuitively, we proposed a method to divide the bifurcation parameter domain into five segments on average and the chaotic domain into 10 segments on average. Then, we calculated the proportion of the number of points in each segment to the total number of points, respectively. The results are shown in Figure 3.

It can be seen from Figure 2 and Figure 3 that 2D-LICM, 2D-ICM, and 2D-MCCM are in chaotic state in the whole parameter domain. However, it is obvious that the bifurcation graph of 2D-MCCM is more uniform, which indicates that its chaotic sequence has strong randomness. At the same time, 2D-MCCM has a larger chaotic interval, which can provide a larger key space for image encryption.

#### 2.2.3. Lyapunov Exponent

The Lyapunov Exponent (LE), which is used to quantitatively describe the speed of adjacent phase points in the phase space at the time of their separation, can illustrate the sensitivity of a chaotic map to initial values. The definition of LE of a 1D discrete map is given in Equation (1) above. Generally speaking, *λ* > 0 means that two adjacent phase points are about to separate, and the chaotic map is in a chaotic state. The larger *λ* is, the faster the adjacent points in the phase space separate from each other and the higher the sensitivity of the initial value is. For an n-dimensional chaotic map, there should be *n* LEs. When more than one Les are larger than 0, the system is in a hyperchaotic state, which means the system has more complex dynamic behavior.

**Figure 4:**
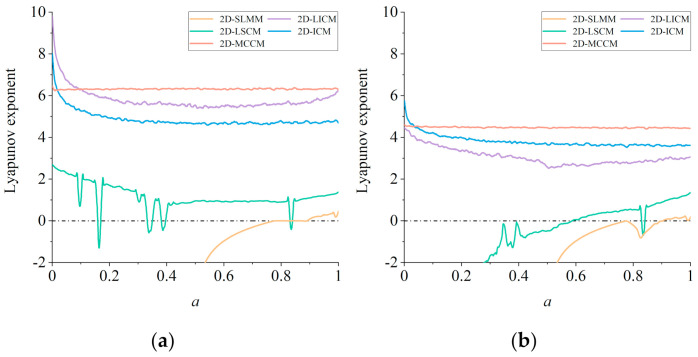
Lyapunov exponent distributions of five 2D chaotic maps: (**a**) comparison of λ_1_ values of five 2D maps; (**b**) comparison of λ_2_ values of five 2D maps.

**Figure 5:**
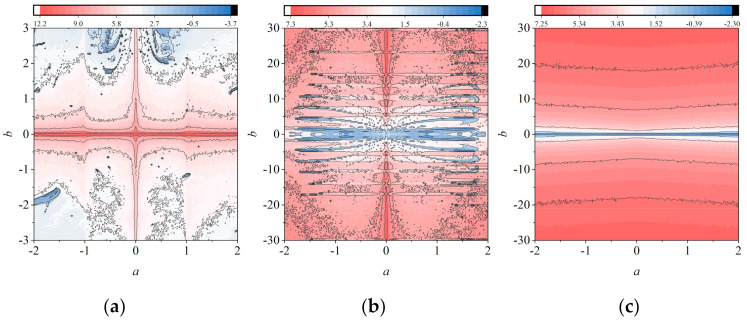
Chaotic diagram of three 2D chaotic maps based on *λ*_1_: (**a**) 2D-LICM; (**b**) 2D-ICM; (**c**) 2D-MCCM.

In general, a 2D chaotic map has two LEs. Figure 4 shows the LEs’ comparison diagram between 2D-MCCM and the four 2D chaotic maps used as comparisons [36]. The parameter *b* of 2D-LICM, 2D-ICM, and 2D-MCCM is set to be 0.5, 21, and 21, respectively, denoting the larger LEs as *λ*_1_ and the smaller LEs as *λ_2_*. It can be seen from Figure 4 that 2D-LICM, 2D-ICM, and 2D-MCCM are all in a hyperchaotic state where *a* ∈ (0, 1]. However, the values of both *λ*_1_ and *λ*_2_ of 2D-MCCM are the largest, indicating that their initial sensitivity is the highest. In order to further study the influence of the values of parameters *a* and *b* on *λ*_1_ of 2D-LICM, 2D-ICM, and 2D-MCCM, we drew a chaotic graph based on *λ*_1_ in Figure 5. The value of *λ*_1_ is larger when the color is closer to red. The closer the color is to red, the greater the value of *λ*_1_ is. Obviously, the red region of 2D-MCCM is the largest, and the value of *λ*_1_ increases with parameter *b*. In addition, parameter *a* does not significantly affect the value of *λ*_1_, indicating that *a* is suitable for image encryption as a key. This shows that 2D-MCCM has the best performance compared with existing 2D chaotic maps and is also suitable for image encryption.

#### 2.2.4. Spectral Entropy

**Figure 6:**
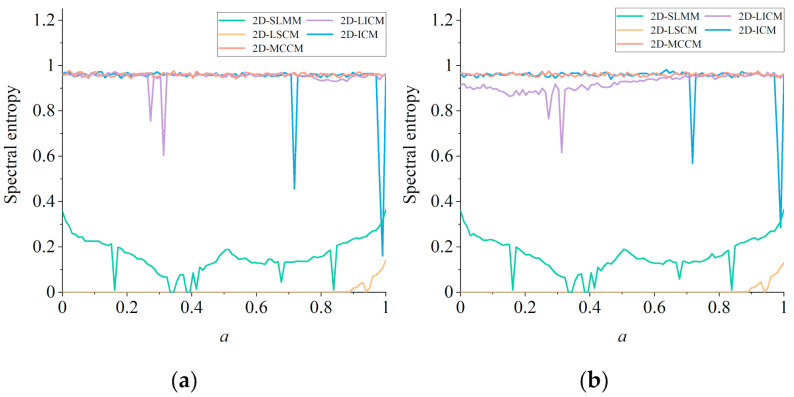
Comparison of spectral entropy of five 2D chaotic map: (**a**) *x* sequence; (**b**) *y* sequence.

Spectral entropy (SE) [37] can be used to quantitatively analyze the similarity between a chaotic sequence and a random sequence. The larger the SE value is, the more similar these two sequences are. The larger the SE value is, the more similar the sequence is with a random one, and the higher its security will be. According to the method in [37], the conditions of SE value of *x* sequence and *y* sequence of each 2D chaotic map are calculated with the transformation of parameter *a*, as shown in Figure 6. It can be seen that compared with the existing 2D chaotic maps, 2D-MCCM can generate chaotic sequences with higher complexity in the whole parameter domain, which can reduce the security risks caused by the reduction of chaos sequence complexity under specific parameters. 

## 3. Design of S-Box

In this section, we devised a simple method of producing an 8-bit S-box using 2D-MCCM and selective self-scrambling.

### 3.1. S-Box Generation

**Figure 7:**
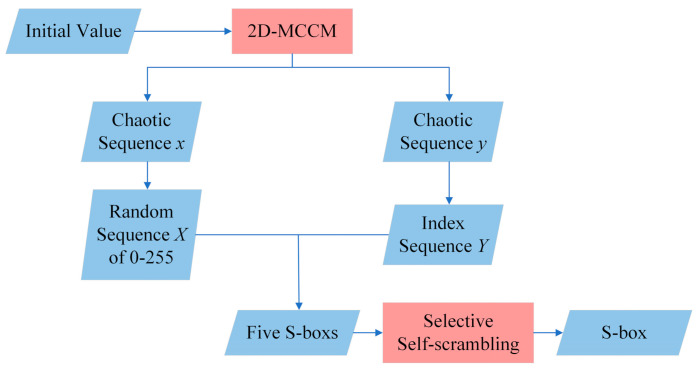
Flow chart of S-box generation algorithm.

Figure 7 briefly illustrates the generation process of an S-box, with specific steps as follows:

1. To set the initial values and parameters of 2D-MCCM.
2. To iterate 2D-MCCM to generate chaotic sequences *x* and *y*.
3. To convert *x* to a random sequence *X* from 0-255 by Equation (8).
  (8) $$X=\left\{floor(x\times 10^{10})\right\}\mathrm{mod}256$$
4. To sort *y* in ascending order and record its position as index sequence *Y*.
5. To select the value in *X* according to *Y* and check whether the value already exists in the S-box. If not, store the value in the S-box until there are 256 non-repeated values in the S-box.
6. Then, to randomly generate four S-boxes according to the method in Step 5.
7. Since linear attack and differential attack are the two most common attack modes, the two S-boxes with the best performance are selected according to the average non-linearity *N_avg_* of S-boxes and the maximum differential approximation probability *DP_max_* of the S-boxes, which are defined as *S_1_* and *S_2,_* respectively. The calculation method is shown in Equation (9), and the larger the value is, the better the performance of the S-box is.
  (9) $$f=\left\{\begin{array}{l} \begin{array}{cc} 1.5N_{avg}-DP_{\max} & D>18 \end{array}\\ \begin{array}{cc} 1.5N_{avg}-2DP_{\max} & D<18 \end{array} \end{array}\right.$$
8. *S_2_* is used to scramble *S_1_* to get the final S-box.

Table 1 shows an 8-bit S-box based on the method above. Since this method only uses chaotic map iteration without complex matrix row and column transformation, it has high generation efficiency. At the same time, there is a selective self-scrambling, which ensures the performance of the generated S-box.

### 3.2. Performance Analysis of S-Box

In order to evaluate the performance of the constructed S-box, the following five methods are used for analysis in this section.

#### 3.2.1. Nonlinearity

As a nonlinear calculation element, the nonlinear degree is an important index to evaluate the performance of the S-box. The expected value of the nonlinear degree is 112. Walsh spectrum [38] is usually used to calculate the nonlinearity of the S-box, which is defined as:

(10) $$N_{f}=2^{-n}(1-\underset{\omega \in GF(2^{n})}{\max}|S_{<f>}(\omega )|)$$

where *GF*(2*^n^*) represents the Galois domain with space size of 2*^n^*, and *S*_< *f* >_(*ω*) is the cyclic spectrum of function *f* (*x*), which is defined as:

(11) $$S_{f}(\omega )=2^{n}\sum_{\omega \in GF(2^{n})}(-1{)}^{f(x)⊕x*\omega }$$

In general, the higher the nonlinearity of an S-box is, the more secure it will be. The eight nonlinearity values of the constructed S-box are 108, 108, 108, 108, 106, 108, and 108, respectively, with an average of 107.75. The minimum nonlinearity of the proposed S-box is easy to be attacked. However, the minimum nonlinearity of the proposed S-box reached 106, which is even better than the average nonlinearity of some S-boxes, indicating that the S-box is capable of resisting a nonlinear cryptanalytic attack.

#### 3.2.2. Strict Avalanche Criterion

The strict avalanche criterion can quantitatively analyze the avalanche effect of Boolean function, that is, when the input of one bit of Boolean function changes, half of the output value will change [39]. For the S-box, the strict avalanche criterion is usually tested by calculating its dependence matrix. If the S-box strictly satisfies the strict avalanche criterion, every element in the dependence matrix will be 0.5.

Based on the method in [40], we calculated the dependence matrix of the S-box, as shown in Table 2. Meanwhile, it can be found that the average deviation of the elements in the dependence matrix from the expected value of 0.5 is 0.0327, which tends to be close to 0 and can satisfy SAC.

#### 3.2.3. Bit Independence Criterion

Bit independence criterion (BIC) is a desirable feature for cipher transformation. For the Boolean functions *f_i_* and *f_j_* with two output bits of the S-box, if *f_i_*⊕*f_j_* is highly nonlinear and satisfies SAC as much as possible, then the S-box satisfies the BIC.

Based on the method in [40], we calculated Bic nonlinearity and BIC-SAC of S-box, as shown in Table 3 and Table 4. It was obtained that the mean value of BIC nonlinearity and BIC-SAC of the proposed S-box is 103.2857 and 0.5008, respectively. And the mean value of BIC-SAC is even better than that of the AES S-box, showing a very outstanding BIC performance.

#### 3.2.4. Differential Approximation Probability

Differential approximation probability (DP) can be used to quantitatively analyze the crypt function’s resistance to differential attack. It represents the maximum probability of the output, which will be Δ*y* in a given Boolean function when the input difference is Δ*x*, and the expected value of *DP* is 0.0156. For an 8-bit S-box, the calculation formula of *DP* is as follows:

(12) $$DP=\underset{\Delta x\neq 0,\Delta y}{\max}(\frac{\#\{x\in X|f(x)⊕f(x⊕\Delta x)=\Delta y\}}{2^{8}})$$

where *X* = {0, 1, ⋯, 255}. For the S-box, the less the maximum *DP* value, the stronger is the ability to resist a differential cipher attack. Table 5 shows the differential approximation matrix of the proposed S-box, and the maximum value is 10. In addition, the maximum *DP* value of the proposed S-box can be calculated from Equation (12) to be 0.0391, which is close to the expected value, indicating its strong resistance to differential attacks.

#### 3.2.5. Linear Probability

Linear Probability (LP) can be used to quantitatively analyze the cryptographic function’s ability to resist a linear attack, and the expected value of *LP* is 0.0625. As a nonlinear element, the S-box can realize the nonlinear map between input and output, and its ability to resist nonlinear attacks is very important. For an 8-bit S-box, the formula of *LP* is as follows:

(13) $$LP=\underset{a_{x},b_{x}\neq 0}{\max}(\frac{\#\{x\in X|x\cdot a_{x}=f(x)\cdot b_{x}\}}{2^{8}}-\frac{1}{2})$$

where *X* = {0, 1, ⋯, 255}, *a_x_* and *b_x_* are input and output masks, respectively. In general, A lower *LP* value shows a stronger S-box of resisting the linear cryptanalysis attacks. Based on equation (13), we calculated the *LP* value of the proposed S-box to be 0.125, which is around the expected value, indicating its strong resistance to linear cryptanalysis attacks.

#### 3.2.6. Performance Comparison

In order to better demonstrate the performance of the proposed S-box, the S-box generated by several recently proposed representative algorithms was analyzed [21,22,23,24,25,26], and the results are shown in Table 6. It can be seen that the proposed S-box was distinguished in all aspect. In addition, it can be concluded that AES S-box was not optimal on BIC-SAC and SAC, indicating that it is very difficult to design an S-box that is optimal in all indicators.

## 4. Proposed Image Encryption and Decryption Algorithm

### 4.1. Image Encryption Algorithm

**Figure 8:**
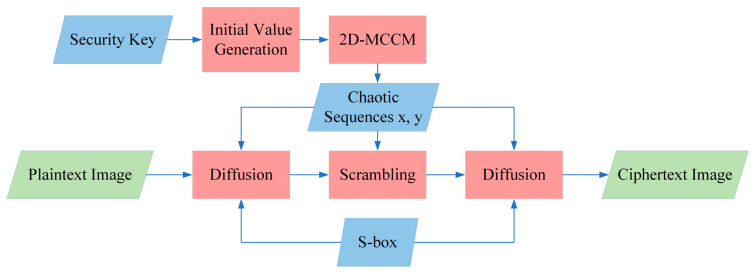
Flowchart of the image encryption algorithm.

In this section, an image encryption algorithm based on 2D-MCCM and a new S-box is proposed. The 2D-MCCM was used to process the key and generate the initial value, and a “diffusion-scrambling-diffusion” framework was used to improve security. Diffusion and scrambling are based on the random sequence generated by 2D-MCCM with both efficiency and security. The proposed S-box was used for the second diffusions, and its nonlinear characteristics were utilized to enhance the sensitivity of the algorithm to small changes in pixels. The algorithm structure is shown in Figure 8.

#### 4.1.1. Initial Value Generation

The selection of the key affects the security of the whole encryption algorithm. In this section, we set the key *K* of the encryption algorithm as 32 random integers of 8 bits with a value range of [0, 255]. At this time, the length of the key *K* reached 256 bits, which is enough to resist violent attacks. For easy calculation, *K* is divided into three parts, such that *K* = [*a*_1_, ⋯, *a*_4_, *b_1_*, ⋯, *b*_4_, *k_1_*, ⋯, *k*_24_]. In addition, in order to enhance the key sensitivity of the algorithm, we used 2D-MCCM in the process of generating initial values from the key. The specific steps are shown in Algorithm 1.
**Algorithm 1 The generation of initial values****Input:** *K*: 32 8-bit random integers with values ranging from [0, 255]. 1: *x_r_ =* [*a*_1_ + *b*_1_ × (*k*_1_ + ⋯ + *k*_6_)]mod *π* − *π*/2; 2: y*_r_ =* [*a*_2_ + *b*_2_ × (*k*_7_ + ⋯ + *k*_12_)]mod *π* − *π*/2; 3: *a_r_ =* [(*k*_13_ + ⋯ + *k*_18_)/*a*_3_
*+* b*_3_*]mod 4 − 2; 4: *b_r_ =* [(*k*_19_ + ⋯ + *k*_24_)/*a*_4_
*+ b*_4_]mod 10 + 20; 5: **for** i = 1 to 10^4^ **do** 6: Iterative 2D-MCCM map with *x_r_*, *y_r_*, *a_r_ and b_r_* as initial values to obtain chaotic  sequences *X* and *Y*; 7: **end for** 8: *Z* = *X* × *Y*; 9: *x* = (|*Z*(1)| + ⋯ + | *Z*(2 × 10^3^)|)mod *π* − *π*/2; 10: *y* = (|*Z*(2 × 10^3^ + 1)| + ⋯ + | *Z*(4 × 10^3^)|)mod *π* − *π*/2; 11: *a_0_ =* (|*Z*(4 × 10^3^ + 1)| + ⋯ + | *Z*(6 × 10^3^)|)mod 4 − 2; 12: *b_0_ =* (|*Z*(6 × 10^3^ + 1)| + ⋯ + | *Z*(10^4^)|)mod 10 + 20; **Output:** Initial value (*x_0_*, *y_0_*, *a*, *b*)

#### 4.1.2. S-Box-Based Diffusion

We designed a novel diffusion method based on 2D-MCCM and S-box, which can magnify small changes in pixels and quickly expand to the entire image. The diffusion algorithm has two steps: forward diffusion and reverse diffusion. Two diffusions in the opposite direction can make the diffusion more sufficient and improve the stability of the algorithm. Forward diffusion and reverse diffusion change the first pixel and the last pixel of the image, respectively, and then spread the transformation to the whole image to change the value of each pixel in the image. The specific process is shown in Algorithms 2 and 3.

##### Forward Diffusion


**Algorithm 2 The forward diffusion process**
**Input:** *P*: The plaintext image of size *M* × *N.* *S*: The proposed S-box. *X*, *Y*: Chaotic sequences generated by iterating 2D-MCCM map. 1: *A*(1) = {*S*(*P*(1) + 1) + floor[*X*(*S*(1) + 1) × 10^5^] + floor[*Y*(*S*(256) + 1) × 10^5^]}mod 256; 2: **for** i = 1 to (*M* × *N −* 1) **do** 3: *k_1_* = *i* mod 256 + 1; 4: *k_2_* = (255 *− i*) mod 256 + 1; 5: *A*(*i* + 1) = {*S*(*A*(*i*) + 1) + *S*(*P*(*i +* 1) + 1) +     floor[*k_1_* × *X*(*S*(*k_1_*) + 1) × 10^5^] + floor[*k_2_* × *Y*(*S*(*k_2_*) + 1) × 10^5^]}mod 256; 6: **end for** 7: *A* = reshape(*A*, *M*, *N*); **Output:**
*A*: The matrix of forward diffusion.

##### Reverse Diffusion


**Algorithm 3 The reverse diffusion process**
**Input:** *B*: The global scrambled image of size *M* × *N.* *S*: The proposed S-box. *X*, *Y*: Chaotic sequences generated by iterating 2D-MCCM map. 1: *C*(*M* × *N*) = {*S*(*B*(*M* × *N*) + 1) + floor[*X*(*S*(256) + 1) × 10^5^] +      floor[*Y*(*S*(1) + 1) × 10^5^]}mod 256; 2: **for** i = (*M* × *N*) to 2 **do** 3: *k_1_* = (255 − *i*) mod 256 + 1; 4: *k_2_* = *i* mod 256 + 1; 5: *C*(*i* − 1) = {*S*(*C*(*i*) + 1) + *S*(*B*(*i* − 1) + 1) +     floor[*k_1_* × *X*(*S*(*k_1_*) + 1) × 10^5^] + floor[*k_2_* × *Y*(*S*(*k_2_*) + 1) × 10^5^]}mod 256; 6: **end for** 7: *C* = reshape(*C*, *M*, *N*); **Output:**
*C*: The matrix of reverse diffusion.

#### 4.1.3. Global Scrambling

A scrambling process can effectively reduce the correlation between pixels and reduce the redundant information in the image. The traditional scrambling method usually scrambles the index matrix of the pseudo-random sequence generated by the chaotic system once, but sometimes it cannot have a good scrambling effect because of the uncertainty of the pseudo-random sequence. For this reason, a new global scrambling method was designed, which gradually scrambles the image from local to global. The specific steps are shown in Algorithm 4.
**Algorithm 4 The global scrambling process****Input:** The image A with size *M* × *N* obtained by diffusion I and the chaotic sequence *X*, *Y*. 1: Divide A into *u* parts on average; 2: *m* = *M*/sqrt(*u*), *n* = *N*/sqrt(*u*); 3: **for** i = 1 to *u*
**do** 4: [~, *I_i_*] = sort[*X*(10^3^ × *i*: 10^3^ × *i* + *m* × *n* − 1)]; 5: **end for** 6: Scrambling each part of A with *I_i_*; 7: [~, *J*] = sort[*Y*(10^3^: 10^3^ + *u* − 1)]; 8: Scrambling the order of the *u* parts with *J* to get a matrix *A*′; 9: *Z* = *X* × *Y*, 10: [~, *K*] = sort[*Z*(10^3^: 10^3^ + *M × N* − 1)]; 11: Scrambling each pixel in *A*′ with *K* to get a matrix *B*. **Output:** The scrambled matrix B.

**Figure 9:**
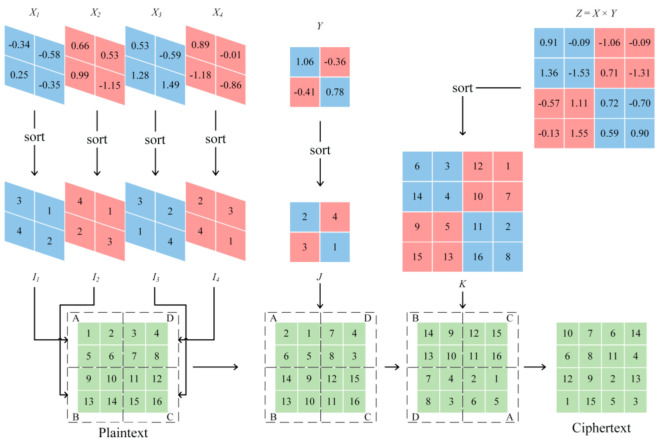
An example of global scrambling using an image of size 4 × 4.

For a more intuitive explanation of the proposed global scrambling method, a numerical illustration for a 4 × 4 image is given in Figure 9. The 4 × 4 plaintext image was evenly divided into four parts for scrambling with a good scrambling effect. Considering safety and efficiency comprehensively, the image was divided into 256 parts for scrambling in practical use.

### 4.2. Image Decryption Algorithm

In simple terms, the decryption process and the encryption process are mutually inverse ones, which mainly include generating initial value from the correct key, finding inverse S-box, inverse backward diffusion, inverse global scrambling, and inverse forward diffusion. For the decryption algorithm proposed, a solution to inverse S-box is a vital step, which remarkably affects the efficiency of the decryption algorithm. The specific generation method is shown in Algorithm 5.
**Algorithm 5 Generation of an inverse S-box****Input:***S*: The proposed S-box. 1: *S*(*S* = 0) = 256; 2: **for** i = 1 to 256 **do** 3: *S*′(*S*(*i*)) = *i*; 4: **end for** 5: *S*′ = reshape(*S*′, 16, 16); **Output:**
*S*′: The inverse S-box.

Since the S-box contains 0 rather than 256, regarding 0 as an index is meaningless. Therefore, before the S-box is used to generate the inverse S-box, 0 is replaced with 256. In this way, pixels with a value of 0 in the ciphertext image can be replaced with 256 to restore the image using the inverse S-box before decryption.

## 5. Simulation Results and Security Analysis

Security is the most important indicator to evaluate the image encryption algorithm. Therefore, the security of the proposed encryption algorithm is evaluated in many perspectives, and comparison with other representative encryption algorithms is made. 

### 5.1. Encryption Result and Histogram Analysis

A histogram of the image can intuitively show the distribution of the pixels. As pixel distribution of each plaintext image is regulated by certain law, the purpose of encryption is to destroy the law, preventing the attacker from acquiring the information in the image histogram, and the pixels should be evenly distributed.

**Figure 10:**
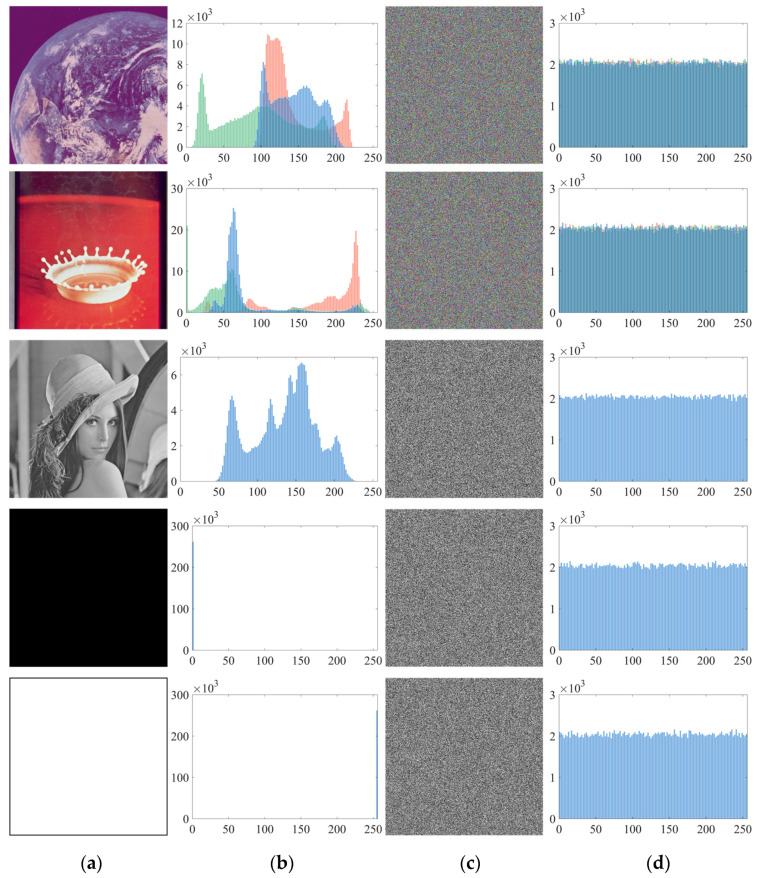
Encryption results and histogram analysis: (**a**) Plaintext images; (**b**) Histograms of plaintext images; (**c**) Ciphertext image; (**d**) Histograms of ciphertext images.

In this section, five 512 × 512 images were used for the experiment. The color images were Earth and Splash, and the gray images were Lena, Black, and White. The results are shown in Figure 10. The histograms of five plaintext images had their own features. After encryption, both grayscale and color images turned into noise-like ciphers. They can only be distinguished by their ciphertext and histogram, and no more useful information can be obtained. This showed that the proposed algorithm can resist the attack of statistical analysis based on pixel distribution.

Since the histogram can only demonstrate pixel distribution, when it comes to the similarity of histograms of the five ciphertext images in Figure 10, it failed to describe their differences accurately. Therefore, the *χ*^2^ statistics [41] is usually used to quantitatively analyze the uniform distribution of pixels in the image, which is defined as:

(14) $$\chi^{2}=\sum_{i=0}^{255}\frac{{\left(f_{i}-g\right)}^{2}}{g}$$

where *f_i_* is the frequency distribution of pixel values in the image and *g* is its theoretical frequency distribution. When the significance level is 0.05, the pixels of the image are considered to be evenly distributed, where point $\chi_{0.05(255)}^{2}$ = 293.2478. In general, the smaller the *χ*^2^ value of an image, the more uniform the pixel distribution is. Table 7 shows the *χ*^2^ test results of the five test images after encryption. It can be seen that the *χ*^2^ value of all ciphertext images was less than 293.2478, indicating that the proposed encryption algorithm had good encryption effect for both color images and grayscale images.

### 5.2. Shannon Entropy Analysis

Shannon entropy can be used to describe the randomness of pixels in an image. The larger its value is, the more similar the image is with a random image. Its calculation method is as follows:

(15) $$H(x)=-\sum_{i=1}^{n}p_{i}\log_{2}p_{i}$$

where *x* is pixel, *p_i_* is probability of taking each pixel, *i* = 1, 2, ⋯, n, 0 < *p_i_* < 1, and *p*_1_ + *p*_2_ + ⋯ + *p_n_* = 1. Shannon entropy for the 8-bit sequences is 8 when all pixels are equally probable. For image encryption, it is desirable to encrypt the image into a random image. Therefore, the closer the Shannon entropy of the ciphertext image is to 8, the better the encryption effect of the algorithm will be.

Table 8 shows the comparison of Shannon entropy of the three gray images before and after encryption. It can be seen that the Shannon entropy of the three ciphertext images was very close to the expected value. For Black and White, although the Shannon entropy was 0 because the pixels in the images were exactly the same, the proposed algorithm still had a good encryption effect after encryption.

Since Shannon entropy is calculated based on the global image, its accuracy and calculation efficiency are easily affected by the image size; thus, it fails to be used as a general test standard. In order to analyze the proposed encryption algorithm more comprehensiv-ely, the local Shannon entropy (LSE) calculation method proposed in [42] was adopted. The LSE is obtained by randomly selecting *k* blocks of the same size from an image and calculating the average entropy of each of them as follows:

(16) $$H_{k,N}(P)=\sum_{i=1}^{k}\frac{H(L_{i})}{k}$$

where *L_i_* is the blocks picked and *k* and *N* are the number of pixels and number of blocks, respectively. In order to facilitate the analysis, the significance level is set at 0.05 and (*k*, *N*) = (30, 1936) during the experiment. At this point, when LSE is within the interval of [7.901901, 7.903037], the image is considered to have passed the test and is close to random distribution.

Twenty-five standard images from the USC-SIPI image database were selected for several experiments. The average was taken as the final result, and the experimental results were compared with three typical algorithms [12,15,30]. The experimental results are shown in Table 9. It can be seen that after encrypting 25 images, 23 ciphertext images of the proposed algorithm passed the LSE test, indicating that the proposed encryption algorithm can encrypt images into ciphertext images with high randomness.

### 5.3. Adjacent Pixel Correlation Analysis

The correlation of adjacent pixels is an important index to evaluate the redundant information in the image, and encryption is to remove the redundant information. The lower the correlation of the adjacent pixels of the ciphertext image is, the less redundant information there is, and the better encryption effect algorithm has. Supposing that *N* adjacent pixels (*x_i_*, *y_i_*) are randomly selected from the image, the calculation method of the correlation of adjacent pixels is as follows:

(17) $$\rho_{xy}=\frac{1}{N}\sum_{i=1}^{N}\frac{\left(x_{i}-\bar{x})(y_{i}-\bar{y}\right)}{\sigma_{x}\sigma_{y}}$$

where *σ_x_* and *σ_y_* are the standard deviations of *x* and *y,* respectively. When the correlation between adjacent pixels in the image is at a low level, its correlation value is close to 0, otherwise it is close to 1. The value of correlation between adjacent pixels can be negative.

**Figure 11:**
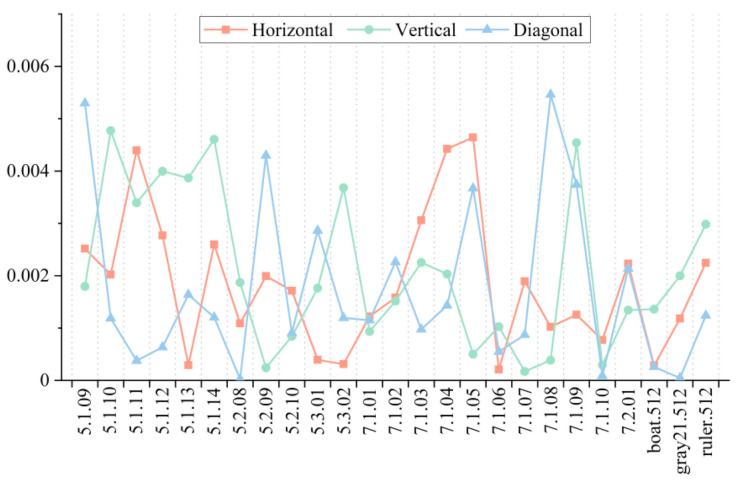
Correlation coefficient of ciphertext images based on proposed encryption algorithm.

In this Section, 25 standard images for encryption and 5000 pairs of adjacent pixels were selected for calculation of the correlation coefficients of the Horizontal, Vertical, and Diagonal diagonals of the ciphertext images. The absolute value of the correlation coefficient was taken, which was convenient for observation, and the results are shown in Figure 11. It can be seen that after the encryption of the 25 standard images, the correlation coefficients of the adjacent pixel points of the ciphertext images in all directions dropped to around 0 and were all less than 0.006, which is approximately no correlation. This shows that the proposed algorithm can significantly disrupt the correlation of adjacent pixels of an image and encrypt arbitrary images of different sizes with excellent results.

**Figure 12:**
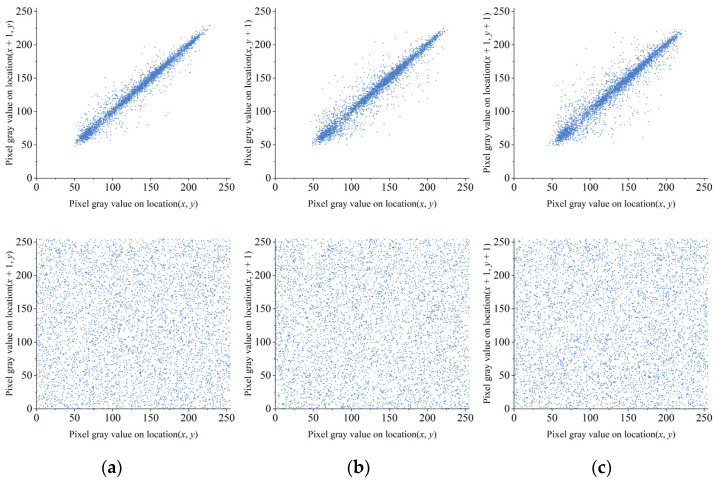
Correlation between adjacent pixels of pre-encrypted and encrypted Image Lena: (**a**) Horizontal; (**b**) Vertical; (**c**) Diagonal.

In addition to the correlation coefficient of the image, we can also analyze the encryption effect of the algorithm using the correlation graph of the adjacent pixels of the image. Figure 12 shows the correlation diagram of adjacent pixels before and after Lena encryption. It can be seen that the pixel pairs of plaintext images were clustered near *x* = *y* and had a strong correlation; however, after encryption, the pixel pairs were evenly distributed and the correlation was greatly reduced. It shows that the proposed algorithm can destroy the correlation between pixels well and has the ability to resist anti-statistics attack.

### 5.4. Robustness to Noise and Data Loss

Due to human or non-human factors, loss and destruction of data may occur in information transmission. An encryption algorithm is considered to be effective if it can recover the plaintext image from the ciphertext image under shear attack or noise attack.

**Figure 13:**
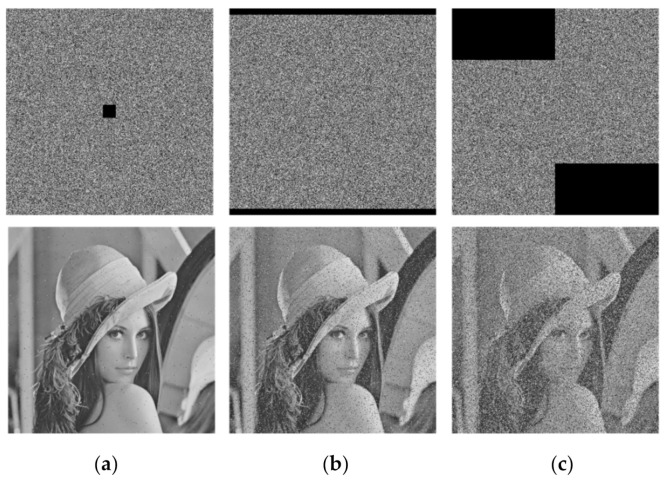
Influence of different proportions of data loss on decryption effects of algorithm: (**a**) 1/256; (**b**) 1/16; (**c**) 1/4.

In this section, we made preliminary analysis of the ability of the proposed encryption algorithm to resist shear attack. We cut down 1/256, 1/16, and 1/4 of the pixels in Lena ciphertext images, respectively, and then decrypted them. The results are shown in Figure 13. It can be seen that even if the ratio of data loss reached 1/4, most information of the image can still be recovered, indicating that the proposed algorithm can resist shear attack.

**Figure 14:**
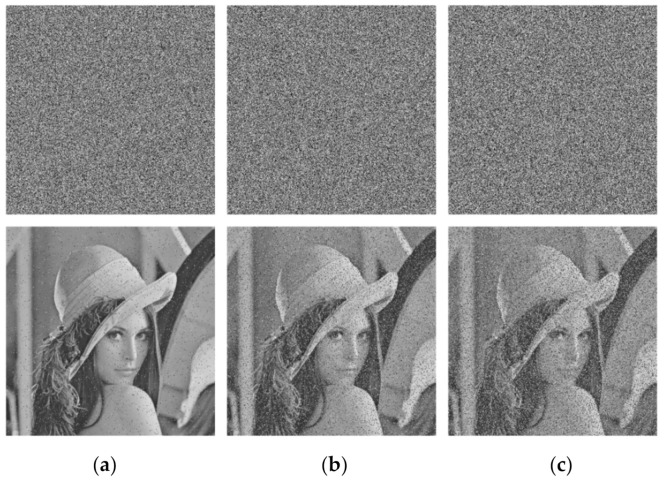
Influence of different proportions of Salt-and-Pepper noise on decryption effects of algorithm: (**a**) 1%; (**b**) 5%; (**c**) 10%.

Subsequently, in order to analyze the robustness of the proposed encryption algorithm to noise attack, we added 1%, 5%, and 10% impulse noise in the ciphertext images of Lena, respectively, and then decrypted them. The results are shown in Figure 14. It can be seen that, although the recovered image information became less and less with the increase of noise ratio, the decrypted images can still be distinguished, indicating that the proposed algorithm is able to resist noise.

### 5.5. Key Sensitivity Analysis

Key sensitivity analysis is an important index to evaluate the security of an encryption algorithm and decryption algorithm, and aims to compare two images obtained by encryption or decryption before and after the slight change of key. The more different they are, the higher the sensitivity of key will be. In practical application, the difference between the two images can be quantitatively analyzed by calculating the rate of change of pixel number (NPCR) and the normalized mean intensity of change (UACI) [43], as shown below.

(18) $$NPCR=\frac{1}{M\times N}\sum_{i=1}^{m}\sum_{j=1}^{n}D(i,j)\times 100\%$$

where

$$D(i,j)=\left\{\begin{array}{l} 1P_{1}(i,j)\neq P_{2}(i,j)\\ 0P_{1}(i,j)=P_{2}(i,j) \end{array}\right.\;UACI=\frac{1}{M\times N}\sum_{i=1}^{m}\sum_{j=1}^{n}\frac{\left|P_{1}(i,j)-P_{2}(i,j)\right|}{F}\times 100\%$$

where *P*_1_ and *P*_2_ are the two images used for comparison, *M* × *N* is the size of the image, and *F* is the maximum pixel value allowed in the image.

In general, the expected values of NPCR and UACI are 99.6094% and 33.4635%, respectively. However, a more rigorous discrimination method is proposed in [43]. That is, when the significance level is *α*, the two images could be considered completely different if the NPCR of the two images is greater than $N_{\alpha }^{*}$ or the UNCI is among the range of $\left[U_{\alpha }^{*-},U_{\alpha }^{*+}\right]$. $N_{\alpha }^{*}$, $U_{\alpha }^{*-}$, and $U_{\alpha }^{*+}$ can be obtained by the following equations:

(19) $$N_{\alpha }^{*}=(F-\phi^{-1}(\alpha )\sqrt{F/M\times N})/(F+1)$$

$$\left\{\begin{array}{l} U_{\alpha }^{*-}=\mu -\phi^{-1}(\alpha /2)\sigma \\ U_{\alpha }^{*+}=\mu +\phi^{-1}(\alpha /2)\sigma \end{array}\right.$$

where

$$\left\{\begin{array}{l} \mu =(F+2)/(3F+3),\\ \sigma =\sqrt{\frac{\left(F+2)(F^{2}+2F+3\right)}{18{\left(F+1\right)}^{2}F(M\times N)}} \end{array}\right.$$

At the same time, $U_{\alpha }^{*+}$ denotes the inverse cumulative density function. In Table 10, the significance level was set at 0. 05 to obtain the expected values of NPCR and UACI for images of different sizes.

**Figure 15:**
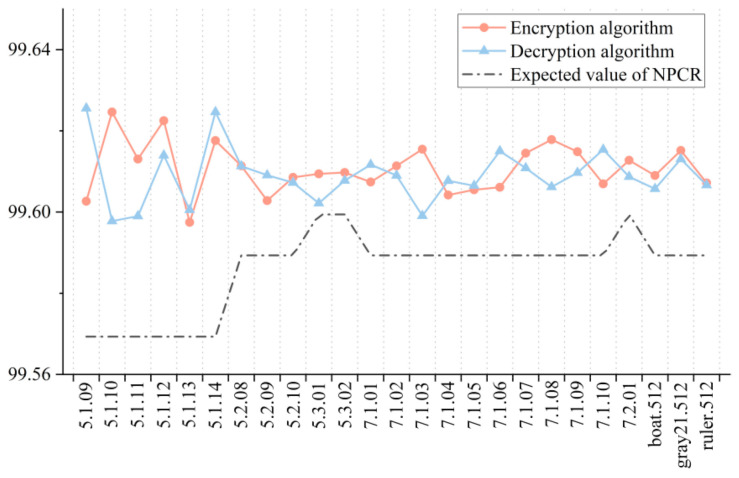
NPCR test results for key sensitivity.

**Figure 16:**
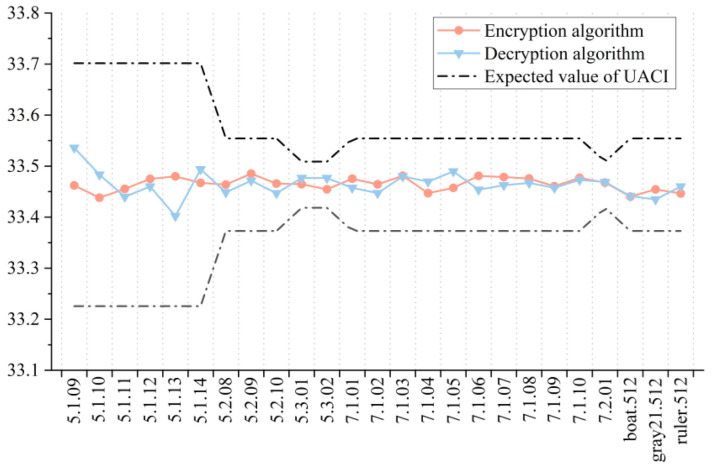
UACI test results for key sensitivity.

In the test of the encryption algorithm, the NPCR value and UACI value between the ciphertext images were obtained after randomly changing the key 1 bit twice. For the decryption algorithm, the correct key was first used for encryption, and then the wrong key was used for decryption. After randomly changing the key 1 bit, the decryption was carried out again, and the image obtained by two decryptions was used for calculation. In order to make the experimental results more accurate and intuitive, we carried out several experiments and took the average values as the final results, as shown in Figure 15 and Figure 16. It can be seen that both the encryption algorithm and the decryption algorithm passed the tests of all 25 images, indicating that they have good key sensitivity.

### 5.6. Difference Analysis

For the encryption algorithm with poor diffusion performance, the attacker can break the encryption algorithm by constructing a specific plaintext image and analyzing the ciphertext image. This attack method is called a differential attack, also known as a selective row plaintext attack. Therefore, it is very important for an encryption algorithm to resist a differential attack.

In general, the stronger the diffusion performance of the encryption algorithm is, the better it is to resist differential attack. Two slightly different plaintext images were encrypted using the algorithm with the same key and then these two ciphertext images obtained were compared to analyze the algorithm’s ability to resist a differential attack, which is called a plaintext sensitivity test.

**Figure 17:**
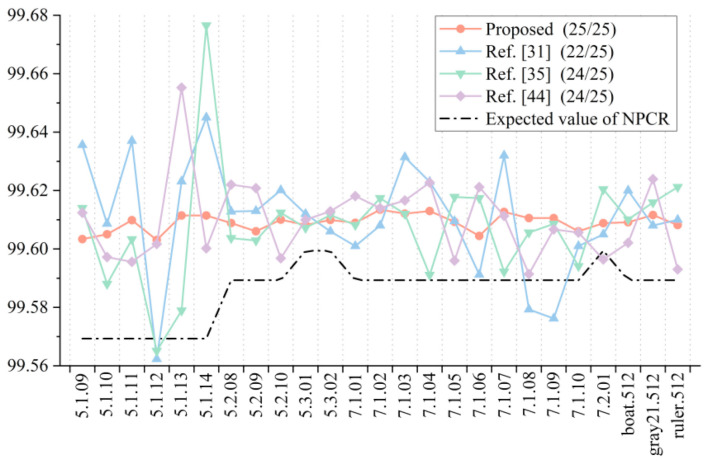
NPCR test results and comparison for plaintext sensitivity.

**Figure 18:**
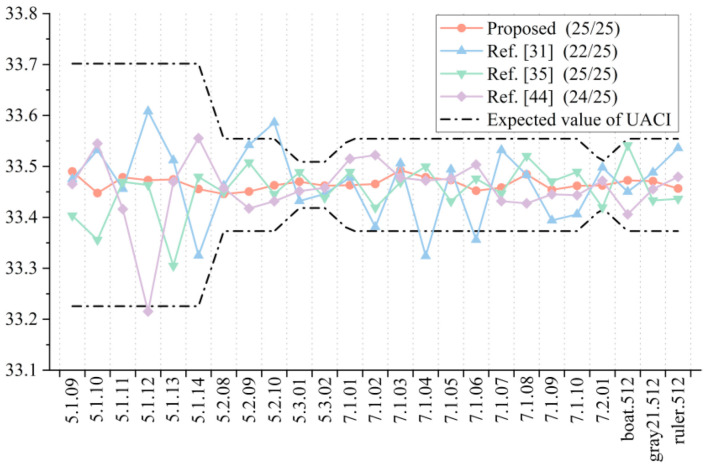
UACI test results and comparison for plaintext sensitivity.

Based on the analysis above, NPCR and UNCI introduced in the previous section were used for the test. Different from the sensitivity of the test key, the 256-bit key *K* was randomly generated firstly, and *K* was used to encrypt the test image. Then, a pixel in the test image was randomly changed by 1, and then *K* was used to encrypt the changed image. Finally, the NPCR value and UACI value of the ciphertext image obtained by two encryptions were calculated and compared with the algorithms in [31,35,44], as shown in Figure 17 and Figure 18. It can be seen that the proposed algorithm passed the NPCR and UACI tests of all 25 images, indicating that the proposed algorithm is superior in resisting a differential attack.

## 6. Conclusions

In this paper, a new 2D chaotic map (2D-MCCM) was proposed. The results of the experiment showed that, compared with existing 2D chaotic maps, 2D-MCCM is very suitable for image encryption due to its advantages in fast iteration speed, large chaotic range, high randomness, stable initial sensitivity, and complexity. Then, we designed a new S-box, which was obtained by selective self-scrambling of multiple initial S-boxes generated by the chaotic sequence of 2D-MCCM. The analysis of the performance of the S-box showed that it has the ability to resist all kinds of security attacks.

Based on 2D-MCCM and the proposed S-box, we designed a new image encryption algorithm, with a main structure of forward diffusion, scrambling, and backward diffusion. The two diffusion processes are based on the nonlinear transformation characteristics of S-box and the chaotic characteristics of 2D-MCCM, which has a good diffusion effect. In addition, scrambling from local to global can effectively reduce the correlation between pixels. Simulation results showed that the algorithm can encrypt various images safely, is superior to several existing algorithms, and has a wide application prospect.

## Figures and Tables

**Table 1:** An example of a generated 8-bit S-box.

*i/j*	1	2	3	4	5	6	7	8	9	10	11	12	13	14	15	16
1	132	88	9	17	218	141	95	86	198	76	136	178	203	128	10	133
2	168	43	72	27	170	222	163	235	142	87	120	217	173	213	179	127
3	34	249	116	192	195	38	254	19	150	175	229	104	177	35	137	162
4	94	176	186	54	211	50	199	220	152	58	45	109	5	231	210	57
5	13	227	64	41	70	48	80	61	167	30	75	91	233	246	172	103
6	191	126	190	62	143	52	135	183	24	215	250	241	53	187	47	112
7	101	147	153	36	145	26	81	130	209	69	248	206	160	55	11	105
8	107	99	129	63	228	92	66	123	223	204	44	102	40	118	7	189
9	93	56	221	1	23	193	207	202	110	100	111	89	244	139	156	208
10	197	67	243	240	242	188	255	6	3	74	219	196	113	185	146	216
11	131	32	73	124	108	122	18	12	205	29	184	245	232	0	166	251
12	85	238	149	230	174	60	140	125	234	154	214	194	90	42	20	253
13	148	225	121	161	82	115	79	114	96	68	97	59	237	98	4	22
14	83	252	158	28	25	138	16	169	212	180	239	201	14	106	2	77
15	31	21	165	65	117	51	49	181	182	144	224	46	236	226	71	15
16	155	119	164	134	159	200	8	247	84	33	151	157	78	39	37	171

**Table 2:** Dependency matrix of the proposed S-box for the strict avalanche criterion (SAC).

*i/j*	1	2	3	4	5	6	7	8
1	0.5313	0.4844	0.4844	0.4844	0.4688	0.4531	0.5469	0.4844
2	0.4844	0.5156	0.4531	0.4844	0.4531	0.4531	0.5156	0.5469
3	0.5469	0.5313	0.4688	0.5156	0.4375	0.5000	0.4531	0.5000
4	0.4844	0.5313	0.4375	0.4531	0.4375	0.4688	0.4688	0.5000
5	0.5313	0.4531	0.4844	0.5313	0.4688	0.4531	0.4219	0.4375
6	0.5000	0.5156	0.5000	0.5313	0.5156	0.4688	0.5938	0.5313
7	0.5000	0.5469	0.5156	0.5313	0.5156	0.4219	0.5469	0.5469
8	0.4844	0.5156	0.5000	0.4219	0.4531	0.4375	0.4844	0.4688

**Table 3:** Bit independence criterion for nonlinearity of the proposed S-box.

*i/j*	1	2	3	4	5	6	7	8
1	-	104	102	106	102	102	102	104
2	104	-	106	104	104	104	104	102
3	102	106	-	106	102	96	100	104
4	106	104	106	-	104	102	108	102
5	102	102	102	104	-	104	102	104
6	102	102	96	102	104	-	106	106
7	102	102	100	108	102	106	-	102
8	104	104	104	102	104	106	102	-

**Table 4:** Bit independence criterion for SAC of the proposed S-box.

*i/j*	1	2	3	4	5	6	7	8
1	-	0.4980	0.5059	0.5176	0.4961	0.4961	0.4746	0.4824
2	0.4980	-	0.5020	0.5313	0.4844	0.5156	0.5039	0.5195
3	0.5059	0.5020	-	0.4980	0.4746	0.4961	0.5176	0.5313
4	0.5176	0.5313	0.4980	-	0.5156	0.4766	0.5039	0.4883
5	0.4961	0.4844	0.4746	0.5156	-	0.4922	0.5078	0.4980
6	0.4961	0.5156	0.4961	0.4766	0.4922	-	0.4883	0.4941
7	0.4746	0.5039	0.5176	0.5039	0.5078	0.4883	-	0.5137
8	0.4824	0.5195	0.5313	0.4883	0.4980	0.4941	0.5137	-

**Table 5:** Differential approximation matrix of the proposed S-box.

*i/j*	1	2	3	4	5	6	7	8	9	10	11	12	13	14	15	16
1	6	8	6	6	6	8	10	10	8	6	8	6	8	6	6	6
2	6	6	6	6	6	8	4	6	6	6	6	6	10	8	8	6
3	6	8	8	8	6	8	6	6	8	6	8	6	6	8	6	6
4	6	8	6	6	6	10	6	6	6	8	6	6	6	6	6	6
5	8	8	8	6	6	6	6	6	8	6	6	8	6	8	6	6
6	6	8	8	6	6	6	6	6	6	6	6	10	6	8	8	6
7	8	8	6	8	6	6	6	8	6	8	6	8	6	8	6	6
8	6	8	6	6	6	8	6	8	8	8	6	6	8	6	6	6
9	6	8	6	8	6	6	6	10	6	6	8	6	6	8	6	6
10	6	6	10	6	8	6	8	8	6	6	6	10	8	6	6	8
11	10	4	6	6	6	8	6	6	6	6	6	6	6	8	6	6
12	6	8	6	8	6	6	8	6	8	6	8	8	6	6	6	6
13	6	6	10	8	6	8	6	8	6	6	6	8	10	6	6	8
14	10	4	8	6	8	8	6	8	6	8	8	6	6	8	8	6
15	6	8	8	8	8	10	8	8	6	6	8	6	6	6	6	8
16	10	6	6	6	6	6	6	6	6	6	8	8	6	8	6	-

**Table 6:** Performance comparison between different S-boxes.

S-Box	Nonlinearity			BIC		SAC			MaxDP	LP
	Max	Min	Avg	BIC-Non.	BIC-SAC	Max	Min	Offset		
Proposed	110	106	107.75	103.29	0.5008	0.5938	0.4219	0.0327	0.0391	0.1250
Ref. [21]	108	102	105.25	103.21	0.5070	0.5938	0.4688	0.0344	0.0547	0.1406
Ref. [22]	108	104	106.5	102.86	0.4939	0.6406	0.4063	0.0276	0.0469	0.1406
Ref. [23]	108	106	106.5	104.07	0.4968	0.6094	0.4219	0.0305	0.0391	0.1328
Ref. [24]	107	101	104.5	103.28	0.5019	0.6094	0.3828	0.0383	0.0391	0.1406
Ref. [25]	108	104	106.25	103.21	0.5004	0.6250	0.3906	0.0347	0.0469	0.1406
Ref. [26]	108	102	104.5	104.64	0.5075	0.6406	0.4219	0.0298	0.0469	0.1172
Ref. [27]	108	104	106.75	103.57	0.5022	0.6250	0.4063	0.0356	0.0391	0.1328
AES	112	112	112	112	0.5011	0.5625	0.4375	0.0295	0.0156	0.0625

**Table 7:** Chi-square test of ciphertext images.

Image			*χ* ^2^
Color image	Earth	R	247.6250
		G	218.9609
		B	254.3496
	Splash	R	223.7773
		G	266.7207
		B	279.7109
Grayscale image	Lena		232.2539
	Black		231.0508
	White		232.4707

**Table 8:** Shannon entropy of plaintext images and ciphertext images.

Image	Plaintext Image	Ciphertext Image
Lena	7.2185	7.9994
Black	0	7.9993
White	0	7.9993

**Table 9:** Comparison of local Shannon entropy of different encryption algorithms.

Image	Size	LSE			
		Ref. [15]	Ref. [30]	Ref. [12]	Proposed
5.1.09	256 × 256	7.902281	7.902967	7.903154	7.902848
5.1.10	256 × 256	7.902198	7.902436	7.901680	7.901973
5.1.11	256 × 256	7.899982	7.904837	7.902725	7.902510
5.1.12	256 × 256	7.902827	7.890333	7.901605	7.902480
5.1.13	256 × 256	7.902281	7.903234	7.901269	7.902726
5.1.14	256 × 256	7.903117	7.902911	7.902341	7.903120
5.2.08	512 × 512	7.902304	7.903500	7.902038	7.902896
5.2.09	512 × 512	7.902022	7.902604	7.902722	7.902585
5.2.10	512 × 512	7.906701	7.902812	7.902478	7.902364
5.3.01	1024 × 1024	7.902119	7.902591	7.902057	7.902349
5.3.02	1024 × 1024	7.902119	7.902850	7.902396	7.902230
7.1.01	512 × 512	7.902191	7.903333	7.902012	7.901949
7.1.02	512 × 512	7.902047	7.901752	7.902484	7.904338
7.1.03	512 × 512	7.902584	7.904041	7.902833	7.902261
7.1.04	512 × 512	7.901913	7.901952	7.902047	7.902943
7.1.05	512 × 512	7.902392	7.902065	7.902568	7.902546
7.1.06	512 × 512	7.902565	7.902507	7.902022	7.902645
7.1.07	512 × 512	7.904015	7.902027	7.902398	7.902925
7.1.08	512 × 512	7.901096	7.902023	7.902137	7.902640
7.1.09	512 × 512	7.902933	7.902044	7.902142	7.902116
7.1.10	512 × 512	7.902534	7.902021	7.902171	7.902623
7.2.01	1024 × 1024	7.902529	7.902051	7.902330	7.902438
boat.512	512 × 512	7.901782	7.902769	7.902046	7.902846
gray21.512	512 × 512	7.902593	7.902200	7.902718	7.902190
ruler.512	512 × 512	7.904102	7.902178	7.902004	7.902658
PASS/ALL	-	18/25	18/25	21/25	23/25

**Table 10:** Expected values of NPCR and UACI for images of different sizes (unit: %).

Image Size	$N_{\alpha }^{*}$	$U_{\alpha }^{*-}$	$U_{\alpha }^{*+}$
256 × 256	99.5693	33.2255	33.7016
512 × 512	99.5893	33.3730	33.5541
1024 × 1024	99.5994	33.4183	33.5088

## Data Availability

Data is contained within the article.

## References

[1] Pereira T., Barreto L., Amaral A. (2017). Network and information security challenges within Industry 4.0 paradigm. Procedia Manuf..

[2] Hua Z., Xu B., Jin F., Huang H. (2019). Image encryption using josephus problem and filtering diffusion. IEEE Access.

[3] Sivakumar T., Venkatesan R. (2015). A novel image encryption using calligraphy based scan method and random number. KSII Trans. Internet Inf. Syst..

[4] Ge R., Yang G., Wu J., Chen Y., Coatrieux G., Luo L. (2019). A novel chaos-based symmetric image encryption using bit-pair level process. IEEE Access.

[5] Zhang L., Zhang X. (2020). Multiple-image encryption algorithm based on bit planes and chaos. Multimed. Tools Appl..

[6] Wang B., Zhang B.F., Liu X.W. (2021). An image encryption approach on the basis of a time delay chaotic system. Optik.

[7] Cao W., Mao Y., Zhou Y. (2020). Designing a 2D infinite collapse map for image encryption. Signal Process..

[8] Fan C., Ding Q. (2018). A novel image encryption scheme based on self-synchronous chaotic stream cipher and wavelet transform. Entropy.

[9] Ding L., Ding Q. (2020). A novel image encryption scheme based on 2D fractional chaotic map, dwt and 4d hyper-chaos. Electronics.

[10] Mondal B., Singh S., Kumar P. (2019). A secure image encryption scheme based on cellular automata and chaotic skew tent map. J. Inf. Secur. Appl..

[11] Niyat A.Y., Moattar M.H., Torshiz M.N. (2017). Color image encryption based on hybrid hyper-chaotic system and cellular automata. Opt. Laser. Eng..

[12] Xian Y., Wang X. (2021). Fractal sorting matrix and its application on chaotic image encryption. Inf. Sci..

[13] Zhou J., Zhou N.-R., Gong L.-H. (2020). Fast color image encryption scheme based on 3D orthogonal Latin squares and matching matrix. Opt. Laser Technol..

[14] Wu J., Liao X., Yang B. (2018). Cryptanalysis and enhancements of image encryption based on three-dimensional bit matrix permutation. Signal Process..

[15] Hua Z., Jin F., Xu B., Huang H. (2018). 2D Logistic-Sine-coupling map for image encryption. Signal Process..

[16] Chai X., Chen Y., Broyde L. (2017). A novel chaos-based image encryption algorithm using DNA sequence operations. Opt. Laser. Eng..

[17] Zhan K., Wei D., Shi J., Yu J. (2017). Cross-utilizing hyperchaotic and DNA sequences for image encryption. J. Electron. Imaging.

[18] Chai X., Gan Z., Yuan K., Chen Y., Liu X. (2019). A novel image encryption scheme based on DNA sequence operations and chaotic systems. Neural Comput. Appl..

[19] Raghuvanshi K.K., Kumar S., Kumar S., Kumar S. (2021). Development of new encryption system using Brownian motion based diffusion. Multimed. Tools Appl..

[20] Kumar M., Kumar S., Das M.K., Budhiraja R., Singh S. (2018). Securing images with a diffusion mechanism based on Fractional Brownian Motion. J. Inf. Secur. Appl..

[21] Hua Z., Li J., Chen Y., Yi S. (2021). Design and application of an S-box using complete Latin square. Nonlinear Dyn..

[22] Hematpour N., Ahadpour S. (2021). Execution examination of chaotic S-box dependent on improved PSO algorithm. Neural Comput. Appl..

[23] Lambić D. (2020). A new discrete-space chaotic map based on the multiplication of integer numbers and its application in S-box design. Nonlinear Dyn..

[24] Özkaynak F., Çelik V., Özer A.B. (2017). A new S-box construction method based on the fractional-order chaotic Chen system. Signal Image Video Process..

[25] Lu Q., Zhu C., Wang G. (2019). A novel s-box design algorithm based on a new compound chaotic system. Entropy.

[26] Ye T., Zhimao L. (2018). Chaotic S-box: Six-dimensional fractional Lorenz–Duffing chaotic system and O-shaped path scrambling. Nonlinear Dyn..

[27] Hu G., Li B. (2021). Coupling chaotic system based on unit transform and its applications in image encryption. Signal Process..

[28] Hua Z., Zhou Y. (2016). Image encryption using 2D logistic-adjusted-Sine map. Inf. Sci..

[29] Zhuang Z.B., Li J., Liu J.Y., Chen S.Q. (2020). Image encryption algorithm based on new five-dimensional multi-ring multi-wing hyperchaotic system. Acta Phys. Sin..

[30] Talhaoui M.Z., Wang X. (2021). A new fractional one dimensional chaotic map and its application in high-speed image encryption. Inf. Sci..

[31] Liu L., Miao S. (2018). A new simple one-dimensional chaotic map and its application for image encryption. Multimed. Tools Appl..

[32] Wolf A., Swift J.B., Swinney H.L., Vastano J.A. (1985). Determining Lyapunov exponents from a time series. Phys. D Nonlinear Phenom..

[33] Di H., Chen H., Ling-Ge J., Hong-Wen Z., Guang-Rui H. (2001). Chaotic characteristics of a one-dimensional iterative map with infinite collapses. IEEE Trans. Circuits Syst. I.

[34] Hua Z., Zhou Y., Pun C.-M., Chen C.L.P. (2015). 2D Sine Logistic modulation map for image encryption. Inf. Sci..

[35] Cao C., Sun K., Liu W. (2018). A novel bit-level image encryption algorithm based on 2D-LICM hyperchaotic map. Signal Process..

[36] Grassberger P., Procaccia I., Hunt B.R., Li T.-Y., Kennedy J.A., Nusse H.E. (2004). Measuring the strangeness of strange attractors. The Theory of Chaotic Attractors.

[37] Sun K.H., He S.B., Yi H., Yin L.Z. (2013). Complexity analysis of chaotic pseudo-random sequences based on spectral entropy algorithm. Acta Phys. Sin..

[38] Sosa P.M. (2016). Calculating nonlinearity of boolean functions with walsh-hadamard transform. UCSB St. Barbar..

[39] Castro J.C.H., Sierra J.M., Seznec A., Izquierdo A., Ribagorda A. (2005). The strict avalanche criterion randomness test. Math. Comput. Simul..

[40] Webster A.F., Tavares S.E., Williams H.C. (1986). On the design of S-Boxes. Advances in Cryptology—CRYPTO ’85 Proceedings.

[41] Zhang Y. (2018). The unified image encryption algorithm based on chaos and cubic S-Box. Inf. Sci..

[42] Wu Y., Zhou Y., Saveriades G., Agaian S., Noonan J.P., Natarajan P. (2013). Local Shannon entropy measure with statistical tests for image randomness. Inf. Sci..

[43] Wu Y., Noonan J.P., Agaian S. (2011). NPCR and UACI randomness tests for image encryption, Cyber Journals: Multidiplinary Journals in ence and Technology. J. Sel. Areas Telecommun..

[44] Hua Z., Zhou Y. (2017). Design of image cipher using block-based scrambling and image filtering. Inf. Sci..

